# Accidental ingestion of an endodontic file: a case report

**DOI:** 10.1186/s13256-022-03363-1

**Published:** 2022-04-14

**Authors:** Ashkun Naderian, Hooman Baghaie, Vysheki Satchithanandha

**Affiliations:** Royal Brisbane and Women’s Hospital, Brisbane, QLD 4029 Australia

**Keywords:** Accidental, Ingestion, Endodontic instrument, Root canal therapy, Case report

## Abstract

**Background:**

Ingestion of dental instruments is rare during dental surgery but can result in serious complications. Here we describe a case in which an endodontic hand file was accidentally misplaced in situ during endodontic (root canal) therapy. Plain radiographs were used to identify its location, and serial imaging was used to monitor passage of the endodontic file through the gastrointestinal tract, and it ultimately passed without intervention. We conclude by describing methods for surveillance and management of ingested dental instruments.

**Case report:**

A 62-year-old Caucasian male presented to the Emergency Department approximately 2 hours after suspected ingestion or inhalation of an endodontic hand file. He had experienced two episodes of excessive coughing and dyspnea while undergoing endodontic therapy, and was promptly referred by his dentist for further investigation. On admission, plain abdominal radiographs confirmed the position of the file in the duodenum, and serial radiographs were used to monitor its transition and clearance through the gastrointestinal tract. During this time, the patient did not demonstrate any clinical signs of bowel perforation, nor was there any radiographic evidence of pneumoperitonium. The patient was discharged after a final radiograph confirmed the absence of the foreign body.

**Conclusion:**

Ingestion and inhalation of dental instruments can be life threatening and should be managed cautiously, with early input from general surgeons, gastroenterologists, or respiratory physicians for possible endoscopic retrieval, emergent laparotomy, or surgical intervention. Imaging studies are useful for discerning the position of the foreign body and to determine whether retrieval is possible, and the management will ultimately depend on the position and characteristics of the foreign body, as well as risk factors from the patient which may increase the likelihood of perforation, obstruction, or impaction.

## Background

Accidental ingestion or inhalation of dental instruments occurs infrequently during dental treatment. Although rare, the consequences can be dramatic; impaction, perforation, and obstruction of the digestive or respiratory tracts are all possibilities [1]. Some instruments, such as endodontic files, feature sharp ends and have a far greater likelihood of causing perforation. We report a case of a patient who accidentally swallowed an endodontic file during root canal treatment of a maxillary molar tooth. Serial radiographic imaging was used to follow passage of the file through the GI tract, and it was ultimately able to pass without the need for further intervention.

## Case presentation

**Fig. 1:**
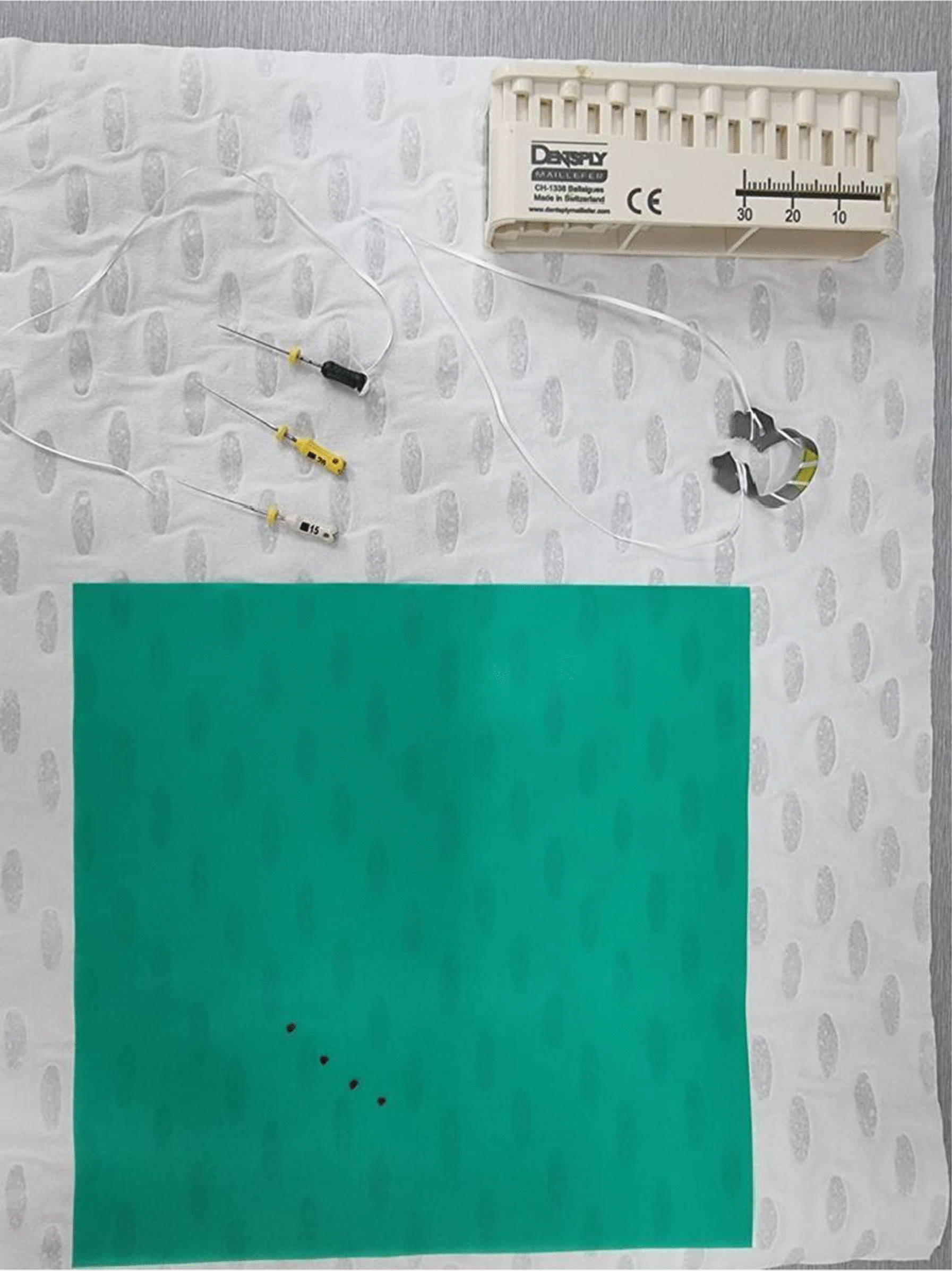
Endodontic files (top left), rubber dam clamp (top right), rubber dam (bottom) [17]

A 62-year-old Caucasian male self-presented to the Emergency Department (ED) at the Royal Brisbane and Women’s Hospital (RBWH) approximately 2 hours after the suspected ingestion or aspiration of an endodontic hand file (Fig. 1) during his visit to the dentist. He was receiving endodontic (root canal) therapy on a maxillary molar tooth. He recalled experiencing two episodes of excessive coughing and dyspnea, lasting several minutes each. Following the coughing, the dentist noted that an endodontic file was missing. He was promptly referred to the ED for investigation of a potentially ingested or aspirated endodontic file. His previous medical history included ischemic heart disease which was managed pharmacologically. Physical assessment revealed stable observations, normal bronchial and vesicular breath sounds with no signs of respiratory distress, cough, dysphagia, abdominal pain, or vomiting. An examination of the oral cavity was normal, suggesting passage of the foreign body beyond the oropharynx.

**Fig. 2:**
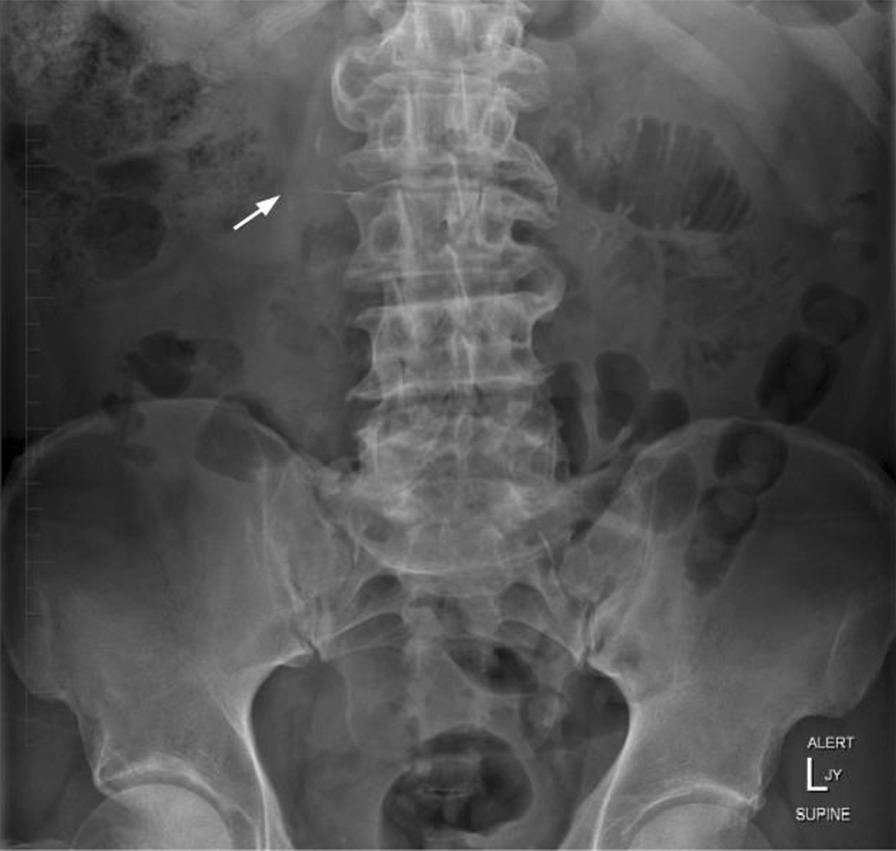
On presentation (2 hours post ingestion). Arrow: the endodontic file can be seen in the duodenum at the level of L2/3

**Fig. 3:**
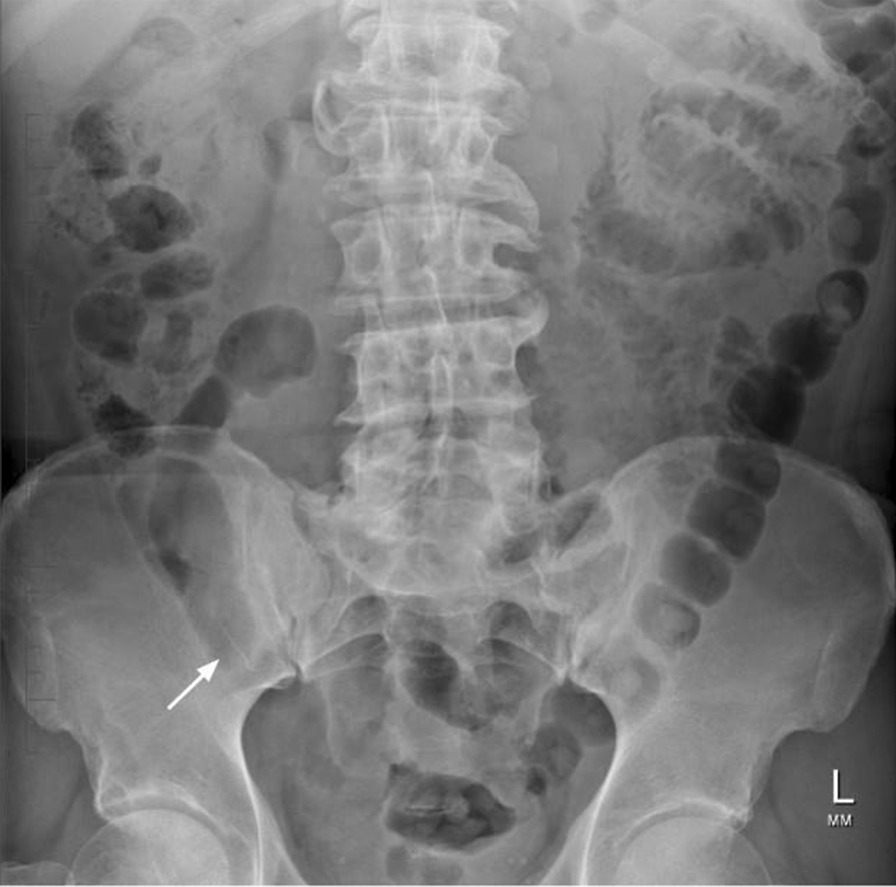
Several hours post presentation. Arrow: the endodontic file has progressed to the distal ileum/ascending colon

Frontal chest and abdominal radiographic imaging confirmed the presence of a 27-mm linear radiopaque foreign body located to the right of the midline of the abdomen, approximately at the level of the L2/3 disc (Fig. 2), representing the endodontic file in the duodenum. General surgical opinion at the time advised inspection and removal via gastroscopy, however by the time this was organized a new abdominal radiograph showed migration of the endodontic file to the distal ileum/ascending colon, beyond the scope of endoscopic retrieval (Fig. 3). The patient was admitted under the care of the general surgery team for observation as an inpatient.

**Fig. 4:**
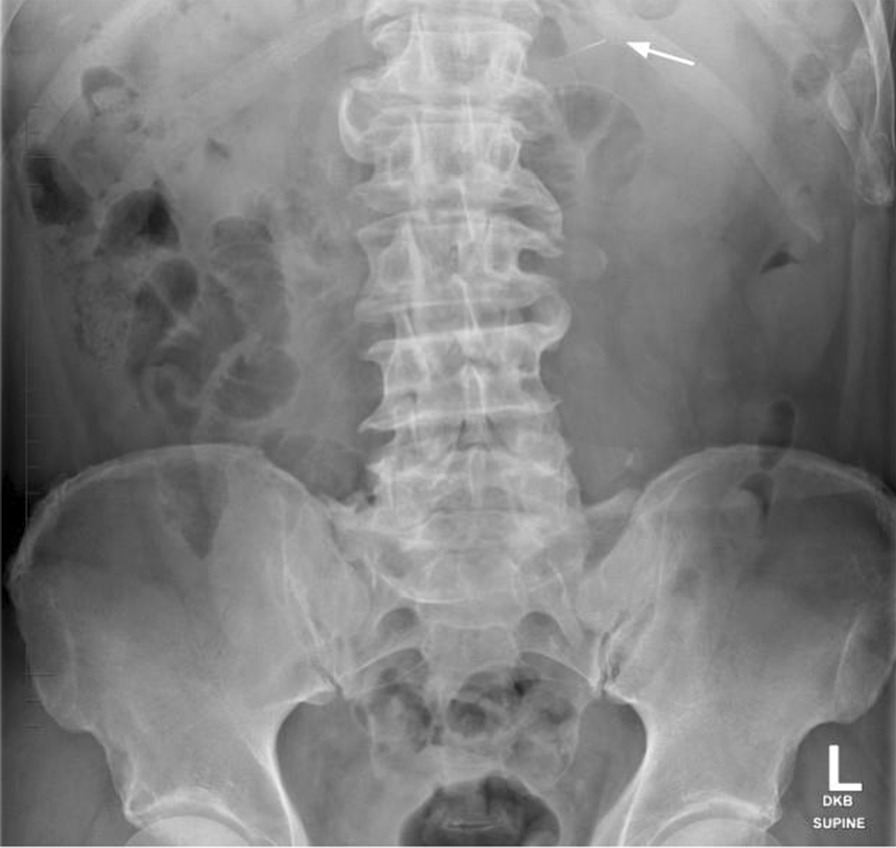
Day 1 post admission. Arrow: the endodontic file has progressed and sits within the transverse colon

Serial abdominal radiographs were used to monitor the migration of the foreign body. A third abdominal radiograph was taken on day 1 post ingestion and depicts a foreign body within the transverse colon (Fig. 4). During this time, the patient did not demonstrate any clinical signs of bowel perforation, nor was there any radiographic evidence of pneumoperitonium. On the second day post ingestion, the endodontic file could no longer be visualized on radiographic imaging, indicating that it had been expelled. The patient was symptom free and was discharged.

## Discussion

Ingestion or aspiration of foreign objects is more common in pediatric patients. It still occurs in adults, however it tends to be accidental or in psychiatric patients. During endodontic therapy, ingestion or aspiration of foreign bodies is vanishingly rare. Retrospective and longitudinal studies have reported incidence rates ranging from 0.00012% to 0.004% [2–4], and the literature around management is sparse.

Once an object passes the tongue, there is a 12:1 chance that it will enter the digestive tract over the respiratory tract [5]. In the gastrointestinal tract, the vast majority of ingested objects pass without intervention, but 10–20% require nonsurgical management and up to 1% require surgical interventions [6]. Important features that impact the management of ingested foreign objects include the patient’s characteristics, object type, and location of object ingested. Generally, ingested objects below 60 mm in length and 25 mm in diameter have a 90% chance of passing through the pylorus and ileocecal valve [6]. Sharper objects such as endodontic files and other dental utensils are more likely to fail to pass the curves of the intestine and can cause esophageal perforation, intestinal puncture, and hemorrhage, all of which may be life threatening [1]. A history of inflammatory bowel disease, strictures, adhesions, hernias, tumors, or diverticula may also increase the risk of impaction and perforation [8].

Patients with suspected inhalation or ingestion of a foreign body should undergo immediate medical assessment. Symptoms of dysphagia, abdominal pain, stridor or wheezing, gagging, choking, neck/throat/chest pain, and most importantly signs of respiratory distress or gastrointestinal obstruction or intestinal perforation should be clarified. The oral cavity and oropharynx should be examined thoroughly for the misplaced object. Once it is clear that the object has passed the oropharynx, radiographic examination is important to determine whether the object has entered the respiratory or gastrointestinal tract. Posteroanterior and lateral chest radiographs as well as an abdominal radiograph are recommended and may aid in detection of complications such as free mediastinal/peritoneal air or subcutaneous emphysema. The majority of dental instruments are radiopaque, however in the case that the object is 4radiolucent, imaging with computed tomography, magnetic resonance imaging, or simply escalation to bronchoscopy, endoscopy, and laparoscopy should be considered based on the clinical picture.

Objects that are lodged in the esophagus should be removed endoscopically, however it is also possible to remove small, blunt objects by passing a Foley catheter beyond the object, inflating the balloon and slowly withdrawing the catheter [9]. Forceps or a forceful cough can be used to remove the object once it reaches the pharynx. Beyond the esophagus, objects which are high risk (sharp or large) may be retrieved endoscopically if the risk of conservative management is deemed too high. Double-balloon enteroscopy has become the standard endoscopic method for retrieving objects in the small bowel, whereas simple esophagogastroduodenoscopy may suffice for objects which are yet to progress beyond the duodenum [10]. If the foreign body has been ingested and is beyond the duodenum, serial radiographic imaging is essential to monitor its passage. Impaction, perforation, or signs of sepsis are all indications for laparotomy or open surgery, but in the absence of these signs the patient can be monitored as an inpatient for spontaneous passage of the foreign body. Conservative outpatient management is an option for asymptomatic patients who have ingested blunt objects less than 60 mm in length and 25 mm in diameter, as these objects are at low risk for perforation or obstruction. These patients should be advised to be aware of the signs of perforation or small bowel obstruction, and to observe their stool continuously. Weekly radiographs may be sufficient in such cases, though failure to pass the object after several weeks is an indication for endoscopic removal or laparoscopy. Patients can generally be safely discharged once the object has been removed or has passed spontaneously.

In contrast to swallowed foreign bodies, inhalation of a foreign body can constitute a medical emergency. Acute dyspnea, asphyxia, laryngeal edema, and cardiac arrest are possible complications [1]. Thin and pointed endodontic instruments also carry the additional risks of perforation and pneumothorax [1]. Immediate retrieval with bronchoscopy is recommended in the case of an aspirated object, with fluoroscopic guidance if the object is difficult to localize. Flexible bronchoscopy has supplanted rigid bronchoscopy as the initial procedure for evaluation and management of aspirated foreign bodies, and has a 90% success rate for retrieval [11]. Chronic retention of objects in the lungs can complicate removal due to the formation of granulation tissue or inflammatory polyps that can obstruct bronchi [12].

**Fig. 5:**
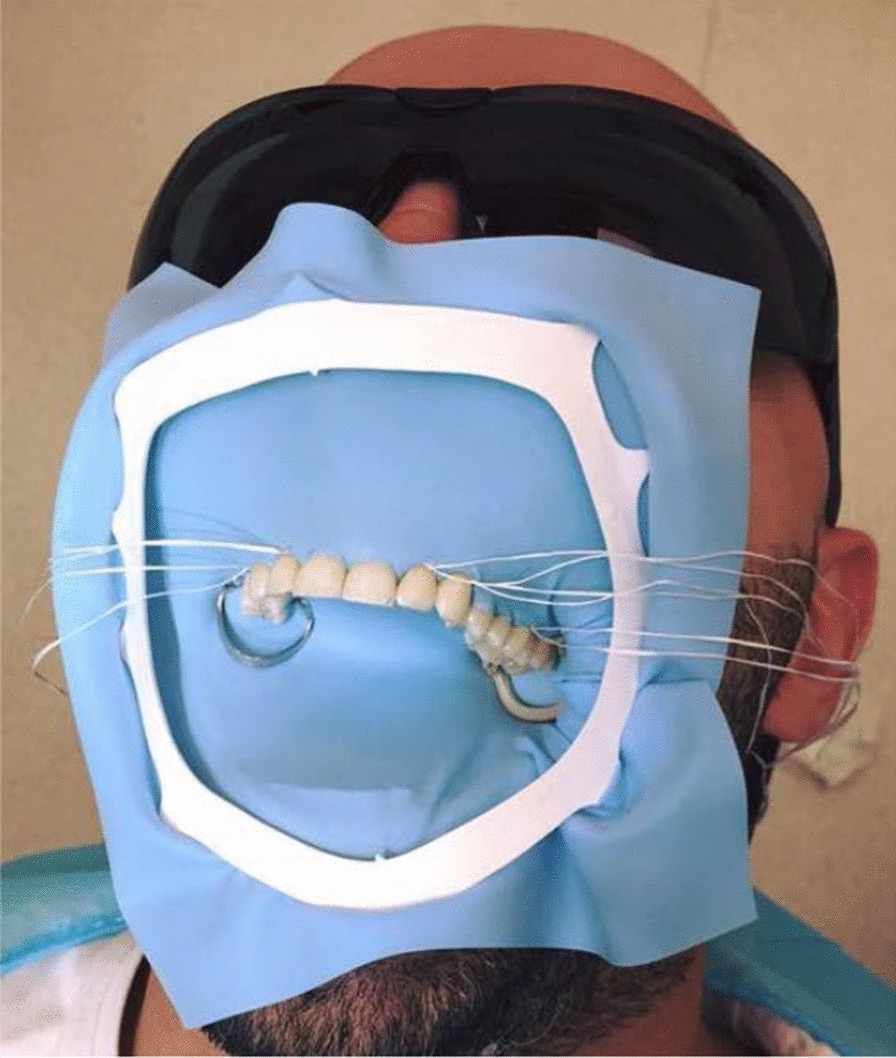
Rubber dam in situ, isolating the upper teeth and covering the oropharynx [18]

Preventative measures during dental procedures can minimize the risk of accidental ingestion or aspiration of objects; covering of the oropharynx, attachment of tools to a length of dental floss material in case they are dropped, adjustment of chair position to sitting over supine, and even the use of magnetic lip retractors are several options. The use of a rubber dam (Figs. 1, 5) during endodontic treatment is mandatory in some parts of the world [13]. The rubber dam isolates the tooth being operated on, and negates the risk of ingestion or aspiration of dental instruments. Surprisingly, a recent survey by Anabtawi *et al.* has shown that only 44% of American general dental practitioners routinely use a rubber dam in endodontic procedures [14]. These findings are paralleled by similar surveys in the UK and New Zealand, with 47% and 57% rubber dam use, respectively [15, 16]. Irrespective, it is useful for the medical practitioner to ask whether rubber dam isolation was used in order to gauge the risk of dental instrument aspiration or ingestion upon presentation.

## Conclusion

Ingestion and inhalation of dental instruments can be life threatening and should be managed cautiously, with early input from general surgeons, gastroenterologists, or respiratory physicians for possible endoscopic retrieval, emergent laparotomy, or surgical intervention. Aspirated objects should be promptly retrieved if this is possible. For ingested objects deemed low risk (blunt objects less than 60 mm in length and 25 mm in diameter), the mainstay of therapy is conservative with serial radiographic monitoring. Higher-risk objects such as endodontic files or other sharp instruments should be retrieved if possible, or monitored vigilantly with routine radiographic imaging if they have progressed beyond the reach of endoscopic retrieval.

## Data Availability

X-ray images are available on the Royal Brisbane and Women’s Hospital PACS system. Patient details and history are available on the Royal Brisbane and Women’s Hospital digital records. There are no other materials or data of note.
